# Umbilical metastasis derived from early stage rectal cancer: a case report

**DOI:** 10.1186/1477-7819-12-82

**Published:** 2014-04-03

**Authors:** Zhigang Zhang, Jianwei Wang, Jian Huang, Xiuyan Yu

**Affiliations:** 1 Department of Oncology, Second Affiliated Hospital, Zhejiang University School of Medicine, Hangzhou 310009, China; 2 Cancer Institute (Key Laboratory of Cancer Prevention & Intervention, National Ministry of Education, Provincial Key Laboratory of Molecular Biology in Medical Sciences), The Second Affiliated Hospital, Zhejiang University School of Medicine, Hangzhou 310009, China

**Keywords:** Rectal cancer, Umbilical metastasis, Sister Mary Joseph’s nodule (SMJN)

## Abstract

**Background:**

Umbilical metastasis, also called Sister Mary Joseph’s nodule (SMJN), is defined as the umbilical nodule associated with advanced metastatic intra-abdominal and pelvic malignancies. A patient with umbilical metastasis has been deemed to have a poor prognosis. Rectal cancer presenting with a SMJN is a rare phenomenon, especially in the early stage and in middle-low rectal cancer.

**Case presentation:**

We report a case of a 70-year-old male presenting with umbilical metastasis derived from rectal cancer (10 cm from the anal verge, T2N0).

**Discussion and conclusion:**

For rectal cancer with umbilical metastasis, the exact metastatic routes as well as the criterion of diagnosis and treatments are not very clear. Here we review the literature on rectal cancer and SMJN to deepen the understanding of this disease.

## Background

Umbilical metastasis, also called Sister Mary Joseph’s nodule (SMJN), is mostly associated with advanced metastatic intra-abdominal and pelvic malignancies [1]. In recent years, there are some sporadic reports of umbilical metastasis. Admittedly, umbilical metastasis implies a poor prognosis but, so far, the primary tumor diagnosis, metastasis pathway and treatment of umbilical metastases are still unclear. Rectal cancer always can be found an abnormal metastasis, like umbilical metastasis [2]. Here, we report a rare case of a rectal cancer with umbilicus metastases and also conduct a substantial review of the literature relevant to umbilicus metastases from colorectal cancer.

## Case presentation

**Figure 1:**
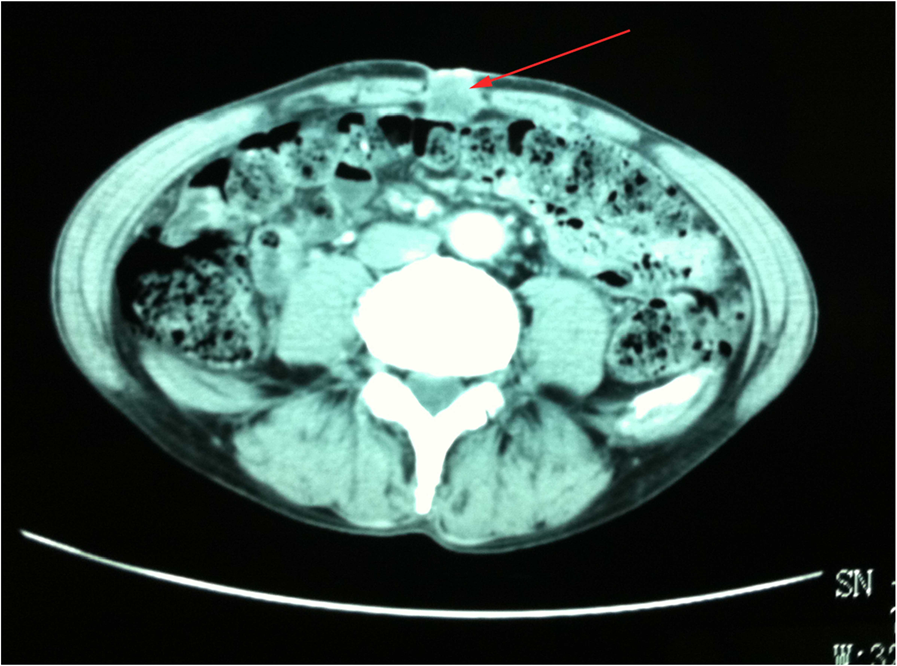
Epigastrium computed tomography: an ill-defined mass at the umbilical area shown by the red arrow.

**Figure 2:**
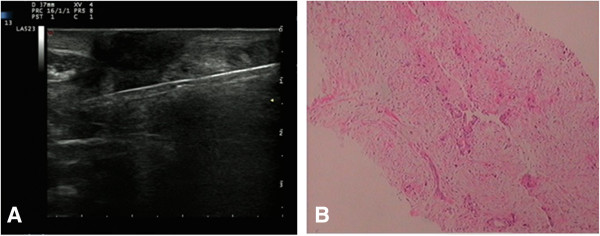
**Core needle biopsy of the umbilical mass: a moderately differentiated adenocarcinoma. A:** Ultrasound-guided core needle biopsy; **B:** Pathological diagnosis for the mass.

**Figure 3:**
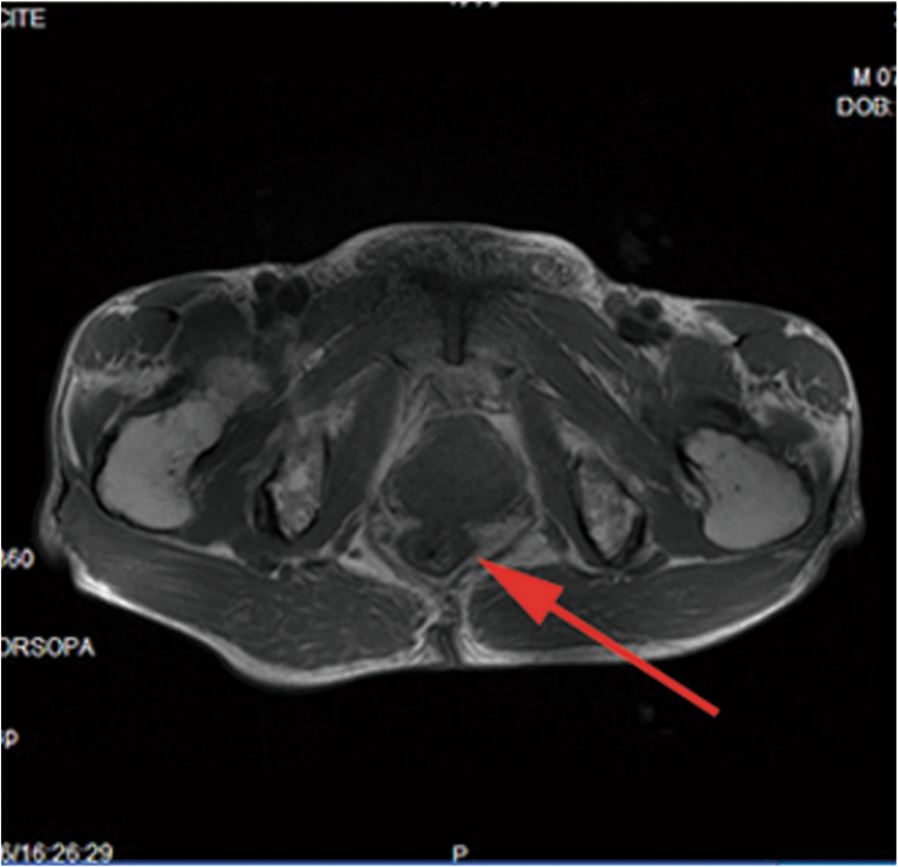
Pelvic enhanced magnetic resonance imaging: a rectal lesion at a distance from the anus of about 86 mm (red arrow).

**Figure 4:**
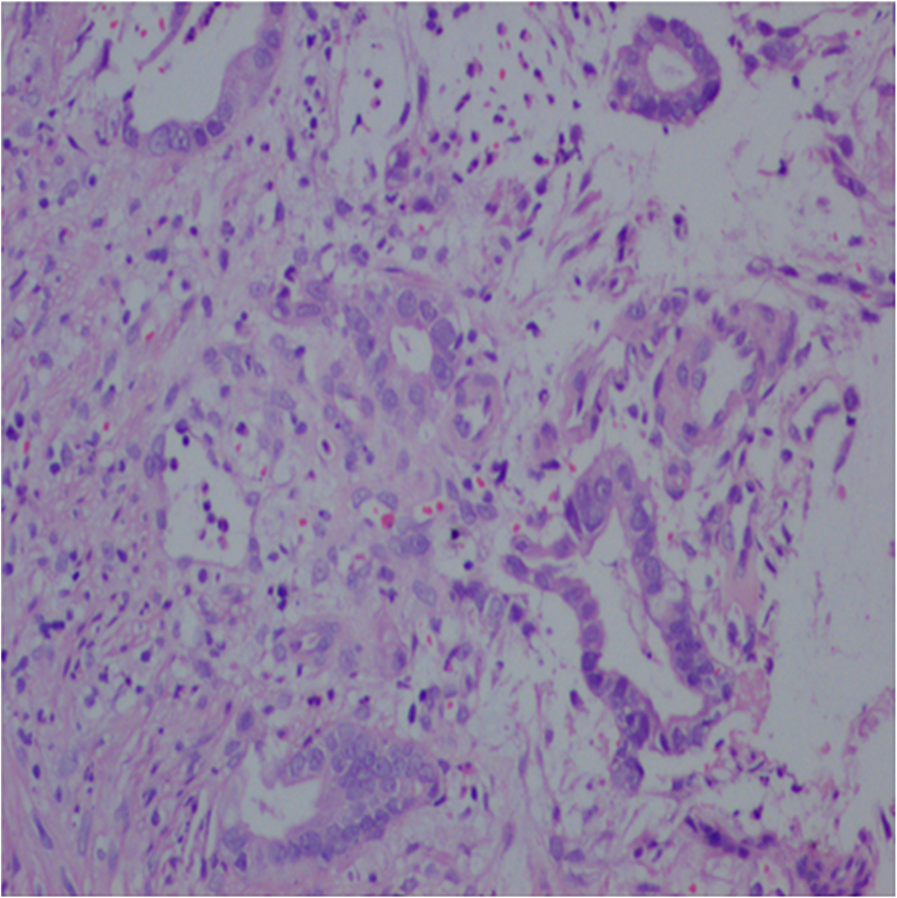
Colonoscopy and histopathology examination: adenocarcinoma of the rectum at 10 cm from the anal verge.

**Figure 5:**
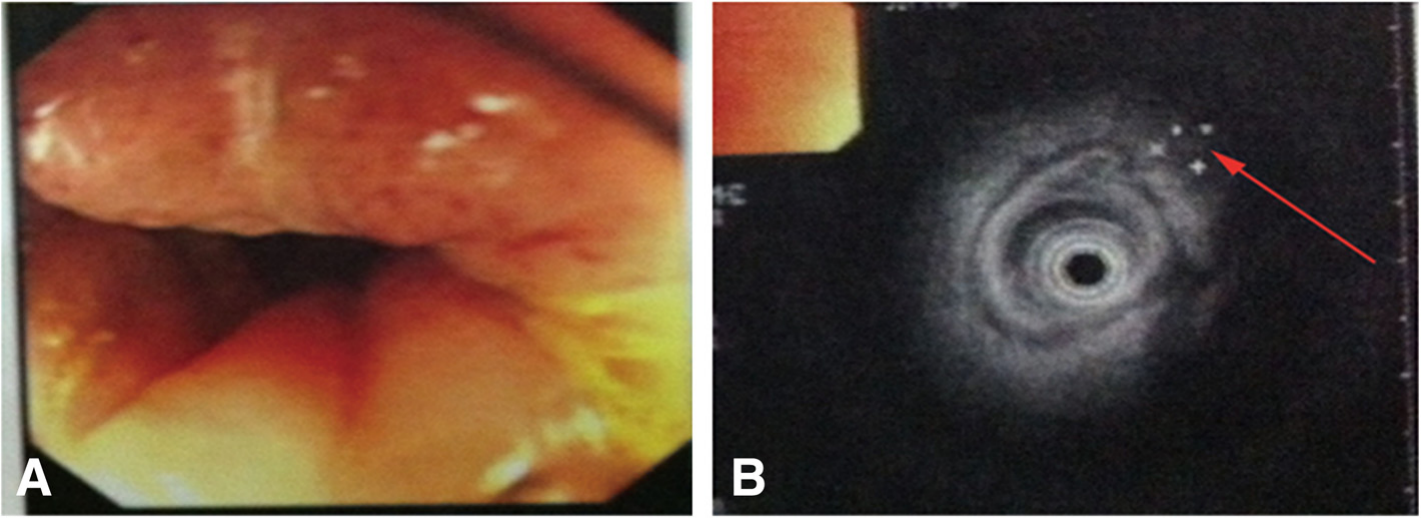
**Colonoscopy and rectal endoscopic ultrasound: rectal tumor infiltrating the muscularispropria (T2N0). A:** Image under colonoscopy; **B:** Rectal endoscopic ultrasound.

**Figure 6:**
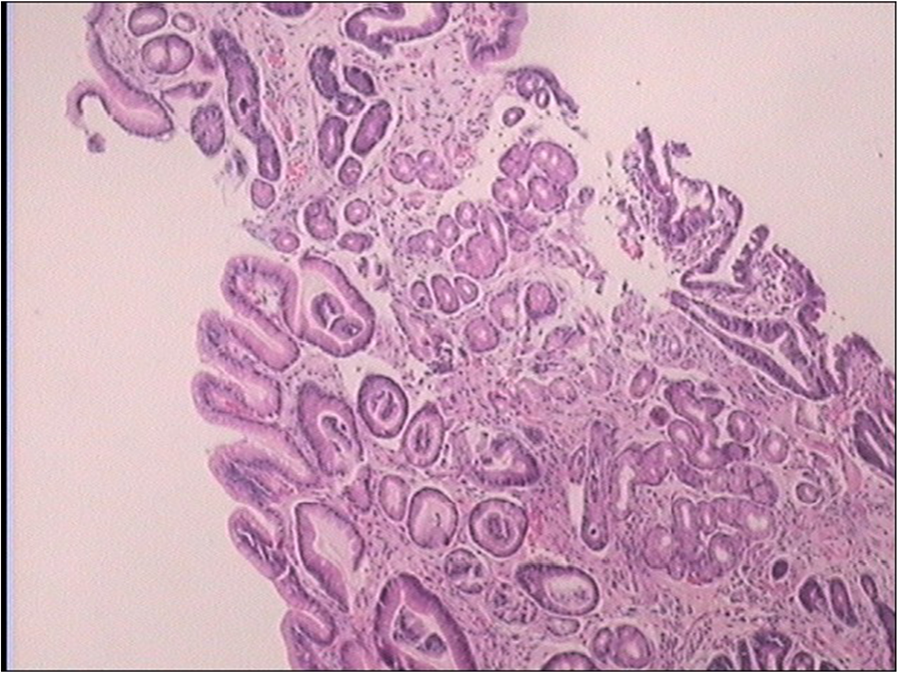
Gastroscope and histopathology examination: a negative result.

In October 2012, a 70-year-old male was admitted to our department complaining of a 5-month history of abdominal pain, weight loss and constipation. Physical examination revealed a hard, craggy, painful, subcutaneous lump, measuring 4.0 × 4.0 cm in the umbilical area of the abdomen. His blood tests are normal except for a markedly elevated carcinoembryonic antigen level (5.4 ng/ml). Epigastrium computed tomography scan showed an ill-defined mass measuring 3.0 × 4.0 cm in diameter in the umbilical area (Figure 1). Core needle biopsy demonstrated a moderately differentiated adenocarcinoma (Figure 2). Pelvic enhanced magnetic resonance imaging revealed anterior rectal wall irregular thickening, gut cavity stenosis, and bowel lesions measuring about 40 mm, with the lesions about 86 mm from the lower edge of the anus (strenghtened on enhanced scanning; Figure 3). Total colonoscopy demonstrated adenocarcinoma of the rectum at 10 cm from the anal verge (Figure 4). Rectal endoscopic ultrasound showed this rectal tumor infiltrating the muscularis propria (Figure 5); according to TNM classification this is a stage I (T2N0) rectal cancer. The patient also underwent a gastroscope (Figure 6) and a chest computed tomography scan, and both were negative. The final diagnosis was rectal carcinoma with umbilical metastasis. After discussion, our mutidisciplinary team considered that this patient should accept surgery and subsequent systemic chemotherapy. The patient refused further treatment and discharged himself from the hospital.

## Discussion

Rectal cancer is one of the most common malignancies around the world, and has an improved prognosis due to the development of diagnostic and therapeutic procedures [3]. However, the survival time could be seriously shortened when distant metastasis occurs. The common distant metastatic sites of rectal cancer are the liver and lung. There are also some sporadic reports showing the spleen, thyroid gland, stomach, pancreas, urinary system and abdominal wall as sites of possible distant metastasis [4]. Though the exact metastasis pathway is not very clear, some potential routes may contribute to it. First, surgery and trauma increase the release of tumor cells into the circulation, and tissue injury has also been documented to promote the growth of implanted tumor cells; these two factors eventually lead to abnormal tumor metastases. A patient with this metastatic pattern usually has a surgical trauma history. Secondly, lymphatic spread is not only towards the adjacent lymph nodes but follows a more diffuse pattern, as the lymph nodes connect to each other. In addition to the lymphatic pipe outside, some other pipes can also can cause tumor metastasis. Thirdly, tumor cells in the blood flow may implant and grow in any proper “soil”. This hematogenous metastatic pattern often involves multiple organs and tissues. Direct extension along the ligaments of embryonic origin, such as the vitelline duct, median umbilical ligament, and falciform ligament, is another possible route of metastatic spread. As in our case, rectal cancer with umbilicus metastases may spread through the urachus.

Umbilical metastasis is also called SMJN, which is defined as a firm umbilical nodule associated with advanced metastatic intra-abdominal and pelvic malignancies. In 1949, Sir Hamilton Bailey first used the name SMJN to describe this umbilical metastatic nodule (in honor of Sister Mary Joseph Dempsey who found the association between umbilical nodules and intra-abdominal malignancy) [1]. Through a review of previous case reports, we found that SMJN arises most frequently from carcinomas of the stomach or ovary, but has also been described with carcinomas of the colon, pancreas, small bowel, gallbladder, lung, prostate, cervix, uterus, fallopian tube, myeloma and mesothelioma [5] (Table 1). Different cancer metastasis to the umbilicus may occur through different routes, which may include the direct extension, lymphatic spread, and tumor implantation.

**Table 1:** Primary site of umbilical metastasis (407 + 58 patients)

**Primary site**	**n (%)**
Stomach	101 (17.9)
Ovary	77 (13.6)
Colon or rectum	66 (11.7)
Pancreas	45 (8.0)
Uterus	26 (4.6)
Gallbladder	12 (2.1)
Cervix	10 (1.8)
Small bowel	11 (1.9)
Breast	8 (1.4)
Prostate	7 (1.2)
Lung	7 (1.2)
Lymphoma	6 (1.1)
Fallopian tube	4 (0.7)
Mesothelioma	4 (0.7)
Esophagus	3 (0.5)
Liver	2 (0.3)
Kidney	2 (0.4)
Bladder	2 (0.4)
Penis	1 (0.2)
Vulva	1 (0.2)
Vagina	1 (0.2)
Myeloma	1 (0.2)
Unknown or others	64 (11.3)

Modified and updated from [5].

SMJN sometimes may be the only symptom present in patients with internal malignancies, and may represent as a late finding in patients with widespread metastases; it not only indicates malignancy but may also indicate poor prognosis. The umbilicus metastases usually occur as the first signal of advanced intra-abdominal malignancy; however, with a lack of understanding, it could be easily mistaken for omphalitis, cyst, hernia and skin disease, which leads to the delayed fiding of primary tumors. This could partly explain why umbilical metastasis patients have a poor prognosis. SMJN should be kept in mind when meeting a patient with an umbilical nodule. Fine needle aspiration cytology (FNAC) could be the best alternative for diagnosis of umbilical nodules, as it has been proved a simple, inexpensive, and reliable technique in making the diagnosis of this mass [6]. The treatments for SMJN should be based on the primary tumor and the patient’s condition. We suggest surgery and systemic chemotherapy, but before surgery we need laparoscopic exploration to estimate the local tumor and abdominal wall, thereby increasing the diagnosis rate and decreasing the rate of the unnecessary “switch operation”.

## Conclusion

Anomalous metastasis is not rare in some malignancies, such as colorectal cancer. Umbilical metastasis from gastrointestinal adenocarcinoma has been reported in many cases, but it is very rare for middle-low rectal cancer. Our report described a case of umbilical metastasis from the early stage rectal cancer (10 cm from the anal verge, T2N0), and we felt regret that the patient did not accept our treatment recommendations. The differential diagnosis of an umbilical lesion should always include metastatic disease apart from benign lesions and primary neoplasms, and FNAC could give us great help to diagnose the lesion. Surgical treatment in umbilical metastasis from gastrointestinal adenocarcinoma is still controversial, but we suggest that surgery and systemic chemotherapy are both necessary for the treatment. Bear in mind that, before surgery, laparoscopic exploration can avoid unnecessary damage.

## Consent

Written informed consent for publication of this case report and the associated images were obtained from the patient.

## Abbreviations

FNAC: fine needle aspiration cytology; SMJN: Sister Mary Joseph’s nodule.

## Competing interests

The authors declare that they have no competing interests.

## Authors’ contributions

ZZ is the first author; XY and JH are the corresponding author of the manuscript. XY and JW collected the patient’s data and provided figures. ZZ, XY and JW were involved in drafting and revising the manuscript. All authors read and approved the final manuscript.
